# LncRNA ROR1-AS1 accelerates osteosarcoma invasion and proliferation through modulating miR-504

**DOI:** 10.18632/aging.103498

**Published:** 2020-12-19

**Authors:** Xiangkun Wu, Lihua Yan, Yongxi Liu, Lilin Shang

**Affiliations:** 1 Department of Orthopaedic Surgery, Nanyang Second People's Hospital, Nanyang 473000, Henan, China; 2 Department of Medical Oncology, Nanyang Second People's Hospital, Nanyang 473000, Henan, China

**Keywords:** osteosarcoma, ROR1-AS1, miR-504

## Abstract

Long non-coding RNAs (LncRNAs) play vital roles in the progression and development of tumors. However, the functional role of ROR1-AS1 in osteosarcoma has not been investigated. We found that ROR1-AS1 was upregulated in osteosarcoma tissues compared to non-tumor samples. Elevated expression of ROR1-AS1 promoted cyclin D1, PCNA and ki-67 expression and increased cell cycle and growth in MG-63 cell. Moreover, overexpression of ROR1-AS1 induced cell migration in MG-63 cell, promoting N-cadherin and vimentin expression and inhibiting E-cadherin expression. Dual-luciferase assay proved that ROR1-AS1 served as one sponge for miR-504 and ROR1-AS1 overexpression suppressed miR-504 expression in MG-63 cell. ROR1-AS1 expression was lower in osteosarcoma tissues compared to non-tumor samples. Pearson's correlation assay showed a negative correlation between miR-504 and ROR1-AS1 expression. MiR-504 overexpression partly abrogated ROR1-AS1-induced effects on osteosarcoma cell migration and proliferation. These data implied that ROR1-AS1 played as an oncogene and might be a new treatment target for osteosarcoma.

## INTRODUCTION

Osteosarcoma was a common type of malignant bone tumor among young adults and teens and originated from specimen of osteoid bon [1–5]. Osteosarcoma is characterized by to high metastasis rate, Although several treatment advancements including postoperative chemotherapy and surgical resection have been attained, overall survival (OS) rate of osteosarcoma cases remains unsatisfactory due to recurrence and metastasis [6–10]. Until now, detailed role and mechanism of metastasis and oncogenesis remained unclear in osteosarcoma [11, 12]. Thus, it is vital to investigate the modulatory mechanism of osteosarcoma and seek new treatment strategies for this disease.

Long non-coding RNAs (LncRNAs) are defined as one type of ncRNAs with the length of over 200 nts and have no ability or limited ability to code protein [13–17]. Emerging references have observed that lncRNAs act as tumor suppressors or oncogenes in various tumors such as lymphoma, nasopharyngeal carcinoma, melanoma, glioma, cholangiocarcinoma, renal carcinoma and osteosarcoma [18–23]. LncRNAs participate in many cellular activities including metabolism, differentiation, metastasis and invasion [24–28]. LncRNA ROR1-AS1 was found to be overexpressed in nasopharyngeal carcinoma, colorectal tumor and bladder cancer [29–34]. However, its role in osteosarcoma and its molecular biological mechanism remain uninvestigated.

We studied the role of ROR1-AS1 in osteosarcoma and identified that ROR1-AS1 was upregulated in osteosarcoma tissues and cells. In addition, ROR1-AS1 overexpression promoted cell growth and migration in osteosarcoma.

## RESULTS

### ROR1-AS1 expression level was upreguated in osteosarcoma tissues

**Figure 1:**
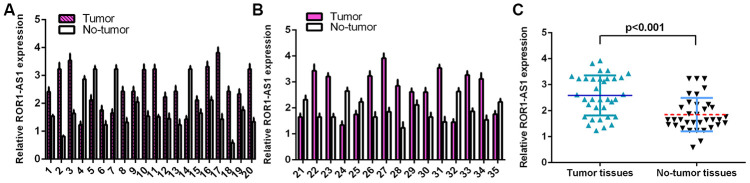
**ROR1-AS1 level in osteosarcoma tissues.** (**A**) The detail expression of ROR1-AS1 of case 1-20 was shown. (**B**) The detail expression of ROR1-AS1 of case 21-35 was shown. (**C**) ROR1-AS1 was higher in osteosarcoma tissues compared to no-tumor samples.

RT-qPCR assay was carried out to measure ROR1-AS1 level in 35 samples. The detailed expression of ROR1-AS1 was shown in Figure 1A and Figure 1B. ROR1-AS1 level was upregulated in 21 cases (26/35, 74.2%) compared to non-tumor samples. Furthermore, ROR1-AS1 expression was higher in osteosarcoma tissues compared to non-tumor samples (Figure 1C).

### ROR1-AS1 promoted cell proliferation in osteosarcoma cells

**Figure 2:**
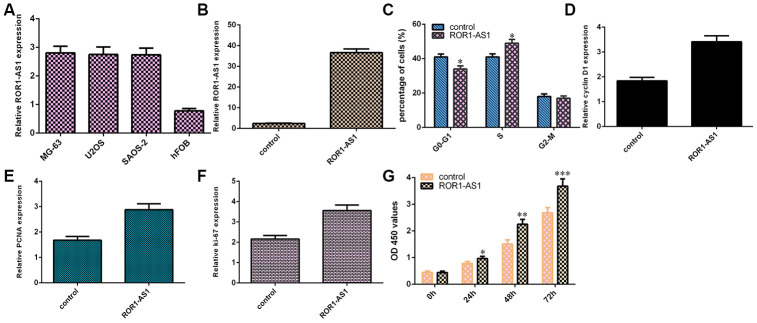
**Gain of ROR1-AS1 promoted cell proliferation of osteosarcoma cells.** (**A**) The ROR1-AS1 expression in osteosarcoma cells (MG-63, U2OS and SAOS-2) and hFOB1.19 was detected by RT-qPCR. (**B**) The expression of ROR1-AS1 in MG-63 cell after transfected with pcDNA- ROR1-AS1 was detected by RT-qPCR. (**C**) ROR1-AS1 overexpression increased cell cycle in the MG-63 cell. (**D**) The expression of cyclin D1 was detected by RT-qPCR. (**E**) The expression of PCNA was measured by RT-qPCR. (**F**) The expression of ki-67 was analyzed by RT-qPCR. (**G**) ROR1-AS1 overexpression induced cell proliferation in MG-63 cell using CCK-8 assay. *p<0.05, **p<0.01 and ***p<0.001.

RT-qPCR showed that ROR1-AS1 expression level was upregulated in osteosarcoma cells (MG-63, U2OS and SAOS-2) compared to hFOB1.19 (Figure 2A). We selected MG-63 cell for function experiments. ROR1-AS1 was overexpressed in MG-63 cell after transfected with pcDNA-ROR1-AS1 (Figure 2B). ROR1-AS1 overexpression increased cell cycle in the MG-63 cell (Figure 2C). Elevated expression of ROR1-AS1 promoted cyclin D1 (Figure 2D), PCNA (Figure 2E) and ki-67 (Figure 2F) expression in MG-63 cell. Moreover, ROR1-AS1 overexpression induced cell proliferation in MG-63 cell (Figure 2G).

### ROR1-AS1 increased cell migration of osteosarcoma cells

**Figure 3:**
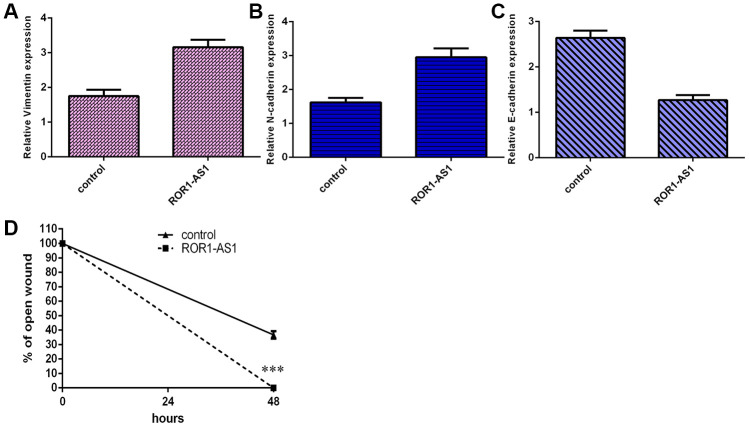
**ROR1-AS1 increased cell migration of osteosarcoma cells.** (**A**) ROR1-AS1 overexpression increased vimentin expression in MG-63 cell. (**B**) Ectopic expression of ROR1-AS1 promoted N-cadherin expression in MG-63 cell. (**C**) The expression of E-cadherin was detected by qRT-PCR. (**D**) The relative wound closure was shown. ***p<0.001.

RT-qPCR data revealed that ROR1-AS1 overexpression increased vimentin expression in MG-63 cell (Figure 3A). Ectopic expression of ROR1-AS1 promoted N- cadherin expression (Figure 3B) and inhibited E-cadherin expression (Figure 3C) in MG-63 cell. Wound healing assay data indicated that elevated expression of ROR1-AS1 increased cell migration in MG-63 cell (Figure 3D).

### ROR1-AS1 functioned as a sponge for miR-504

**Figure 4:**
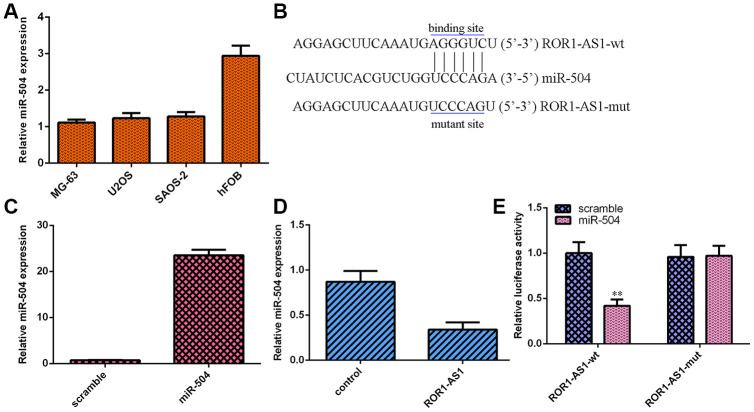
**ROR1-AS1 functioned as one sponge for miR-504.** (**A**) The miR-504 expression in osteosarcoma cells (MG-63, U2OS and SAOS-2) and hFOB1.19 was detected by RT-qPCR. (**B**) ROR1-AS1 has potential binding sites of miR-504 by searching Starbase. (**C**) The miR-504 expression was detected in MG-63 cell after transfection with miR-504 mimic. (**D**) Ectopic expression of ROR1-AS1 suppressed miR-504 expression in MG-63 cell. (**E**) Luciferase activity was impaired in plasmids cotransfected with ROR1-AS1-wt and miR-504 but no change in plasmids cotransfected with ROR1-AS1-mut and miR-504. **p<0.01.

RT-qPCR showed that miR-504 level was downregulated in osteosarcoma cells (MG-63, U2OS and SAOS-2) compared to hFOB1.19 (Figure 4A). ROR1-AS1 has potential binding sites of miR-504 by searching Starbase (Figure 4B). MiR-504 expression was upregulated in MG-63 cell after transfected with miR-504 mimic (Figure 4C). Ectopic expression of ROR1-AS1 suppressed miR-504 expression in MG-63 cell (Figure 4D). To verify hypothesis, dual luciferase analysis was employed. Luciferase activity was impaired in plasmids cotransfected with ROR1-AS1-wt and miR-504 but no change was observed in plasmids transfected with ROR1-AS1-mut and miR-504 (Figure 4E).

### MiR-504 expression level in osteosarcoma tissues

**Figure 5:**
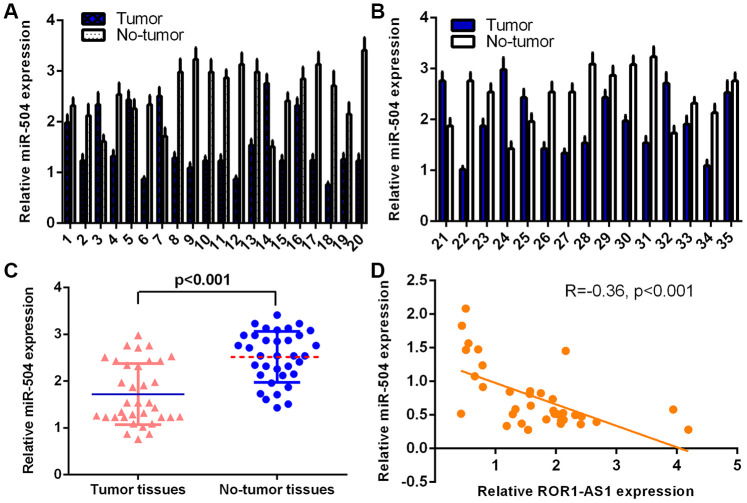
**miR-504 levels in osteosarcoma tissues.** (**A**) The detail expression of miR-504 of case 1-20 was shown. (**B**) The detail expression of miR-504 of case 21-35 was shown. (**C**) ROR1-AS1 was lower in osteosarcoma tissues compared to no-tumor samples. (**D**) Pearson's correlation assay showed one negative correlation between miR-504 and ROR1-AS1 in osteosarcoma tissues.

RT-qPCR assay was carried out for measuring miR-504 level in 30 samples. The detailed expression of miR-504 was shown in Figure 5A and 5B and miR-504 level was downregulated in 21 cases (27/35, 77.1%) compared to non-tumor samples. Furthermore, ROR1-AS1 expression was lower in osteosarcoma tissues compared to non-tumor samples (Figure 5C). Pearson's correlation assay showed a negative correlation between miR-504 and ROR1-AS1 expression (Figure 5D).

### MiR-504 overexpression partly abrogated ROR1-AS1-induced effects on osteosarcoma cell migration and proliferation

**Figure 6:**
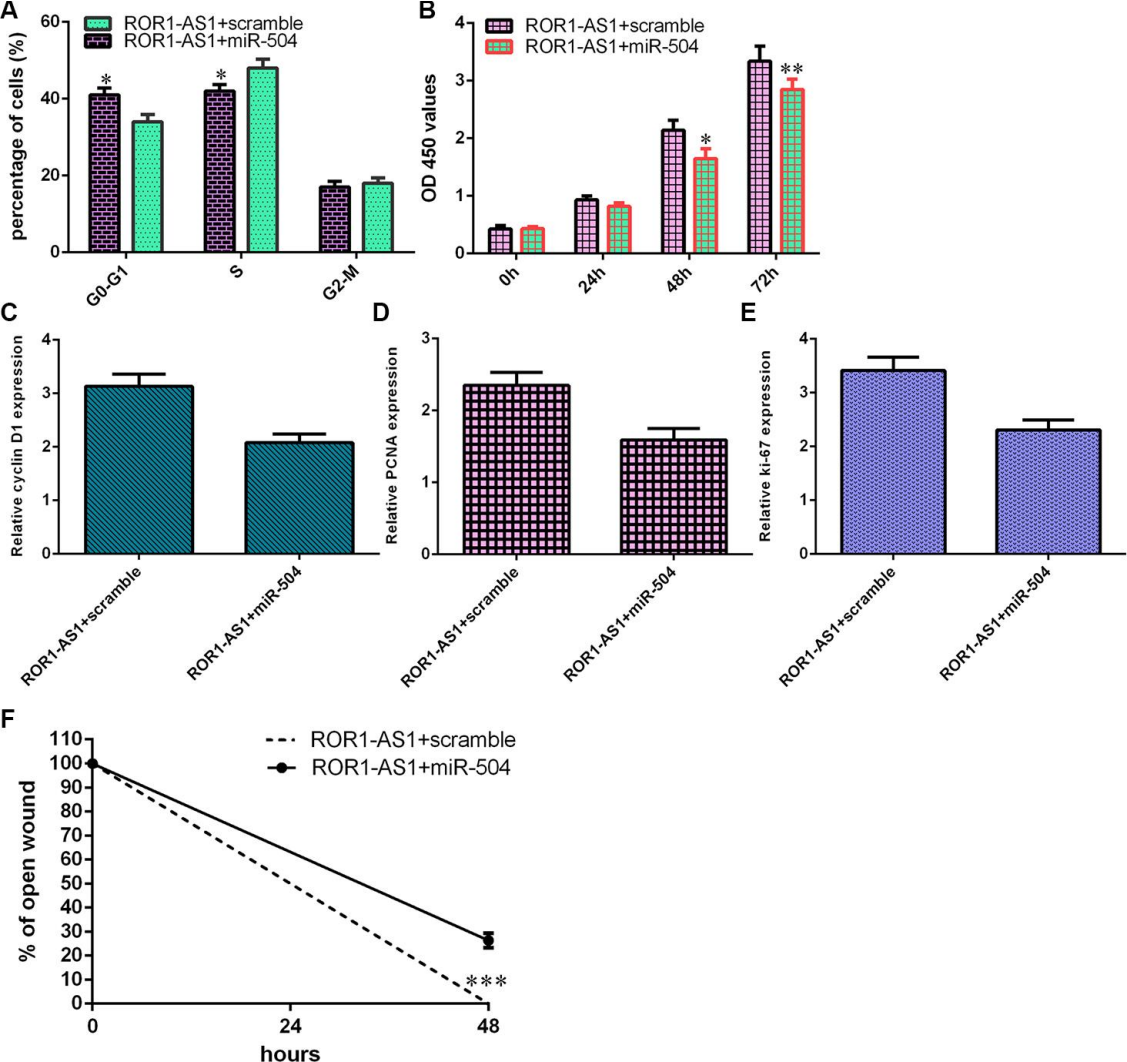
**miR-504 overexpression partly abrogated ROR1-AS1-induced effects on osteosarcoma cell migration and proliferation.** (**A**) Overexpression of miR-504 inhibited cell cycle in ROR1-AS1-overexpressing MG-63 cell. (**B**) Cell proliferation was measured by CCK-8 analysis. (**C**) The expression of cyclin D1 was detected by RT-qPCR. (**D**) The expression of PCNA was measured by RT-qPCR. (**E**) The expression of ki-67 was analyzed by RT-qPCR. (**F**) The relative wound closure was shown. *p<0.05, **p<0.01 and ***p<0.001.

Rescue tests were conducted to study whether ROR1-AS1 regulated proliferation and migration through modulating miR-504 in osteosarcoma cell. Overexpression of miR-504 inhibited cell cycle in ROR1-AS1-overexpressing MG-63 cell (Figure 6A). Ectopic expression of miR-504 suppressed cell growth in ROR1-AS1-overexpressing MG-63 cell (Figure 6B). Elevated expression of miR-504 inhibited cyclin D1 (Figure 6C), PCNA (Figure 6D) and ki-67 (Figure 6E) expression in ROR1-AS1-overexpressing MG-63 cell. MiR-504 overexpression suppressed cell migration in ROR1-AS1-overexpressing MG-63 cell (Figure 6F).

## DISCUSSION

Numerous references have indicated that lncRNAs play vital roles in several biological processes such as tumor initiation and growth [23, 35, 36]. We identified that ROR1-AS1 expression was higher in osteosarcoma tissues compared to non-tumor samples. Elevated expression of ROR1-AS1 promoted cyclin D1, PCNA and ki-67 expression and increased cell cycle and growth in MG-63 cell. Moreover, overexpression of ROR1-AS1 induced cell migration in MG-63 cell, promoting N-cadherin and vimentin expression and inhibiting E-cadherin expression. Dual-luciferase assay proved that ROR1-AS1 functioned as a sponge for miR-504 and ROR1-AS1 overexpression suppressed miR-504 expression in MG-63 cell. ROR1-AS1 expression was lower in osteosarcoma tissues compared to non-tumor samples. Pearson's correlation assay showed a negative correlation between miR-504 and ROR1-AS1 expression. MiR-504 overexpression partly abrogated ROR1-AS1-induced effects on osteosarcoma cell migration and proliferation. These data implied that ROR1-AS1 played as an oncogene and might be a new treatment target for osteosarcoma.

ROR1-AS1 was located in the 1p31.3 and was one novel identified lncRNA in lymphoma [30]. Growing evidences have showed that ROR1-AS1 acts as oncogenes in some tumors including nasopharyngeal carcinoma, bladder cancer and colorectal cancer [32–34]. ROR1-AS1 expression was upregulated in nasopharyngeal carcinoma samples and ROR1-AS1 knockdown inhibited cell invasion and migration and EMT progression in nasopharyngeal carcinoma cell via regulating miR-375 [33]. Chen et al [34]. found that ROR1-AS1 was overexpressed in bladder tumor samples and associated with lymph node metastasis, advanced stage and histological grade. ROR1-AS1 knockdown decreased bladder tumor cell migration and proliferation. Wang et al. [32] indicated that ROR1-AS1 expression was upregulated in colorectal cancer samples and ROR1-AS1 downregulation suppressed cell invasion and migration in colorectal cancer cell through regulating miR-375. However, the functional role of ROR1-AS1 in osteosarcoma remains uninvestigated so far. We observed that ROR1-AS1 expression was higher in osteosarcoma tissues compared to non-tumor samples and ROR1-AS1 overexpression induced osteosarcoma cell migration and proliferation.

Compelling reports demonstrated that lncRNAs might play their roles via regulating miRNAs expression [37, 38]. For instance, LINC01278 induced osteosarcoma progression through sponging miR-133a-3p expression [39]. Gui et al [40]. showed that lncRNA CDKN2B-AS1 knockdown inhibited osteosarcoma migration and proliferation through regulating miR-4458 expression. Hou et al [41]. demonstrated that lncRNA SNHG14 induced osteosarcoma cell invasion, proliferation and migration through modulating miR-433-3p expression. Huang et al [42]. indicated that knockdown of LncRNA FTX inhibited osteosarcoma migration and growth through regulating miR-320a expression. Recently, Chen et al [34]. indicated that ROR1-AS1 expression induced bladder tumor cell migration and proliferation through regulating miR-504. ROR1-AS1 has potential binding sites of miR-504 by searching Starbase. Dual-luciferase assay was conducted to prove that ROR1-AS1 functioned as one sponge for miR-504 and ROR1-AS1 overexpression suppressed miR-504 expression in MG-63 cell. ROR1-AS1 expression was lower in osteosarcoma tissues compared to non-tumor samples. Pearson's correlation assay showed a negative correlation between miR-504 and ROR1-AS1 expression. MiR-504 overexpression partly abrogated ROR1-AS1-induced effects on osteosarcoma cell migration and proliferation.

In summary, we identified that ROR1-AS1 expression was overexpressed in osteosarcoma tissues and cell and ROR1-AS1 overexpression promoted cell migration, EMT progression and migration via modulating miR-504. Thus, ROR1-AS1 played as one oncogene and might be a new treatment target for osteosarcoma.

## MATERIALS AND METHODS

### Specimens, cell culture and transfection

Human osteosarcoma specimens along with normal adjacent specimens were obtained at our hospital. This research was conducted according to Declaration of Helsinki and approved with Nanyang hospital. Each case provided consent signed forms. Cell lines of osteosarcoma (SAOS-2, U2OS, MG-63 and HOS) and osteoblast cell line (hFOB1.19) were utilized in our reference. pcDNA-ROR1-AS1, miR-504 mimic, pcDNA-control and scramble were getting from GenePharma (Shanghai, China). Cell transfections were performed by Lipofectamin3000 (Invitrogen Inc.)

### qRT-PCR

RNA was collected from specimens or cultured cells with TRIzol (TaKaRa, Dalian). qPCR was utilized to quantify miRNA, lncRNA and mRNA expression with SYBR kit (Takara, Dalian) on ABI 7900 qPCR system (Applied Biosystems, USA). 2^-ΔΔCt^ way was used to study relative expression. Primers were utilized as following: ROR1-AS1, 5’-CTGAC GAAAC ACTGG AACTC-3’; 5’-GTCTG ATTTG GTAGC TTGGA TG-3’; GAPDH, 5’-CCAAA ATCAG ATGGG GCAAT GCTGG-3’; 5’-TGATG GCATG GACTG TGGT CATTC A-3’; miR-504 5’-GCTGC TGTTG GGAGA CC-3’; 5’-GCCCT CTGTA TGGGA AAC-3’; U6 5’-CTCGC TTCGG CAGCA CATA-3’; 5’-ACGCT TCACG AATTT GCGT-3’.

### Cell growth and migration assay

CCK-8 analysis was utilized to detect cell growth. Transfected osteosarcoma cells were cultured in the plate of 96-well and 10 μL CCK-8 kit was cultivated with cells for 2 hours. Absorbance at the 450 nm was calculated by microplate reader. Wound healing analysis was utilized to study cell migration. Cells were plated in dish of 6-well and continued to confluence. Pipette tip was used to scratch cell wound and cells were plated in serum-free medium. Relative wound distance was recorded by microscope (Olympus).

### Luciferase assay

To set up luciferase reporter constructs, ROR1-AS13-WT and ROR1-AS13-Mut were cloned into pGL3 plasmids (Promega, WI). Cell transfection was carried out with ROR1-AS13-WT, miR-504 mimic or ROR1-AS13-Mut and scramble with Lipofectamin3000 (Invitrogen Inc.). Luciferase activities were measured using one luciferase reporter system (Promega).

### Statistical analysis

Statistical assay was carried out by SPSS (19.0, SPSS Inc, USA). Data were presented as mean ±standard deviation. Student’s t-test was utilized to determine significant differences. The value of < 0.5 was supposed to be significant.
